# Sulfated alginate oligosaccharide exerts antitumor activity and autophagy induction by inactivating MEK1/ERK/mTOR signaling in a KSR1-dependent manner in osteosarcoma

**DOI:** 10.1038/s41389-022-00390-x

**Published:** 2022-04-13

**Authors:** Zhen Pan, Xiao-juan Wei, Shi-jie Li, Hua Guo, Zhao-hui Li, Ke-ke Zhang, Qian-qian Lyu, Wei-zhi Liu, Qing-cheng Yang, Dong-dong Cheng

**Affiliations:** 1 Department of Orthopedics, Shanghai Jiao Tong University Affiliated Sixth People’s Hospital, 200233 Shanghai, China; 2 Institute of Microsurgery on Extremities, Shanghai Jiao Tong University Affiliated Shanghai Sixth People’s Hospital, 200233 Shanghai, China; 3 Department of Orthopaedic Surgery, Yangpu Hospital, Tongji University School of Medicine, 200090 Shanghai, China; 4 MOE Key Laboratory of Marine Genetics and Breeding, College of Marine Life Sciences, Ocean University of China, 266003 Qingdao, China; 5 Laboratory for Marine Biology and Biotechnology, Qingdao National Laboratory for Marine Science and Technology, 266235 Qingdao, China

**Keywords:** Bone cancer, Chemotherapy

## Abstract

Alginate oligosaccharide (AOS) has the function to inhibit tumor progression and the sulfated modification can enhance the antitumor activity. To date, the function and mechanism of sulfated AOS (AOS-SO_4_) in tumors remain largely elusive. We prepared AOS by the enzymatic degradation of alginate, collected AOS-SO_4_ by sulfating following the canonical procedure. Using these materials, in vitro assays showed that both AOS and AOS-SO_4_ elicited antitumor effects in osteosarcoma cells. Sulfated modification significantly enhanced the antitumor activity. In addition, AOS-SO_4_ had obvious effects on cell cycle arrest, apoptosis, and autophagy induction in vitro and in vivo. Mechanistically, we observed that AOS-SO_4_ treatment triggered proapoptotic autophagy by inhibiting MEK1/ERK/mTOR signaling. The ERK activator reversed AOS-SO_4_-induced autophagy. More importantly, we found that KSR1 interacted with MEK1 and functioned as a positive regulator of MEK1 protein in osteosarcoma cells. High KSR1 expression was significantly associated with poor survival in osteosarcoma patients. Together, these results suggest that AOS-SO_4_ has a better antitumor effect in osteosarcoma by inhibiting MEK1/ERK/mTOR signaling, which is KSR1-dependent; thus, AOS-SO_4_ can be a new potential therapeutic candidate for the treatment of osteosarcoma.

## Introduction

Osteosarcoma is the most frequent primary bone tumor in children and adolescents. The overall incidence is 3.4 per million per year worldwide [1]. According to Surveillance, Epidemiology and End Results–US National Cancer Institute (SEER) statistics, new incidents have been rising by 0.4% per year in the last decade [2]. With the introduction of neoadjuvant chemotherapy, standard multiagent chemotherapy combined with surgical resection yields long-term survival rates of 70% [3]. However, during the past three decades, the survival rate has basically progressed slowly [4]. Metastatic disease either at diagnosis or at the time of recurrence portends a poor prognosis with a survival of 20–30% [5, 6]. For the treatment of osteosarcoma, multidrug resistance is a common problem, and there has been no successful targeted treatment of osteosarcoma. Therefore, it is urgent to optimize treatment strategies and develop new therapeutic agents for osteosarcoma patients.

Alginate is a natural carbohydrate polymer and consists of β-D-mannuronic acid and α-L-guluronic acid linked via 1,4-glycosidic bonds [7]. Alginate exists widely in various brown seaweeds. Alginate and alginate-derived polymannuronate (PM) and polyguluronate (PG) can be further depolymerized into AOS with various structures through enzymatic digestion, acid hydrolysis, and oxidative reductive free-radical depolymerization [8]. AOS, as a water-soluble functional oligomer, has been regarded as a non-toxic and biodegradable polymer. Furthermore, AOS displays a wide variety of physiological activities such as immunomodulatory [9], neuroprotective [10], anti-inflammatory [11], antioxidant [12], and antitumor [13] effects. Alginate oligosaccharide (AOS degree- polymerized, DP = 2–10) has been demonstrated to attenuate the progression of human prostate cancer cells through the suppression of the Hippo/YAP/c‐Jun pathway [14]. In addition, Chen [15] reported that AOS (DP = 2–5) could suppress osteosarcoma progression and might be used as a potential drug for osteosarcoma therapy. Researchers report that sulfated oligosaccharides have better antitumor activity. The sulfated oligosaccharide from sargassum fusiform has been found to have the ability to enhance antitumor activities in Sarcoma 180 tumors [16, 17]. Whether sulfate modification can improve the antitumor activity of AOS has not been reported. Thus, in this study, we wanted to compare the antitumor activity of AOS and AOS-SO_4_ and investigate the underlying mechanism of AOS-SO_4_.

Autophagy is a highly conserved catabolic process that includes the formation of phagophores and autophagosomes and fusion with lysosomes [18, 19]. The role of autophagy in tumor progression is complex. Several studies have suggested that apoptosis and autophagy are interrelated and affect each other [20, 21]. In recent years, several natural organic compounds have been reported to exert their anticancer activities by inducing autophagy in tumor cells [22, 23]. MEK/ERK is an important pathway that promotes cell survival and is involved in the regulation of autophagy in mammalian cells [24, 25]. ERK and its upstream kinase MEK enhance autophagy by promoting the production of the Beclin1 protein [26]. mTOR kinase inhibits multiple autophagy-promoting proteins via phosphorylation and has been shown to be the most crucial effector and therapeutic target [27]. Some pathways, including the Akt and MEK/ERK signaling pathways, can activate mTOR kinase and inhibit autophagy [28]. KSR1, as a crucial scaffolding protein, has been shown to promote cell proliferation and oncogenic potential by enhancing signaling activation through the RAS/RAF pathway [29]. Several studies have shown that KSR1 acts downstream of RAS as a molecular scaffold for the Raf/MEK/ERK kinase cascade and interacts with MEK to promote the activation of MEK and ERK [30]. To date, the function of KSR1 in osteosarcoma remains unknown, especially whether KSR1 plays a critical role in AOS-SO_4_-mediated antitumor activity.

Therefore, according to previous research on the antitumor effect of AOS and the relationship between the occurrence of osteosarcoma and autophagy, we reasonably speculated that AOS-SO_4_ had better antitumor activity and that AOS-SO_4_ inhibited the progression of osteosarcoma by inducing autophagy. Here, our study will demonstrate the antitumor effect of AOS-SO_4_ on osteosarcoma and find the internal connection between AOS-SO_4_ and autophagy.

## Results

### Characterization of AOS

**Fig. 1:**
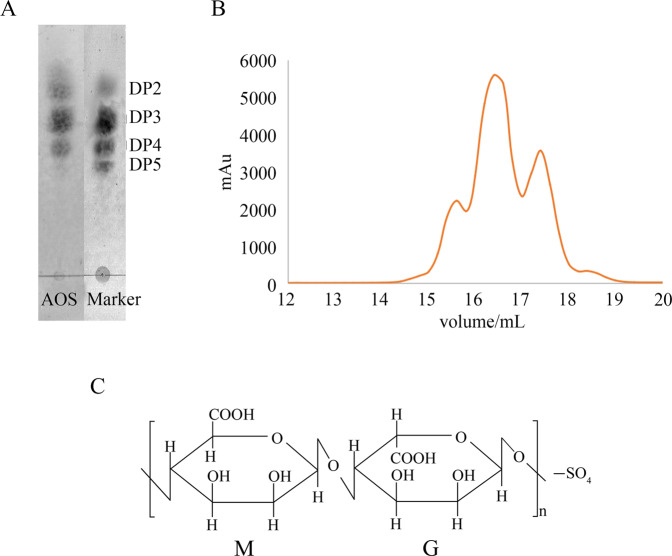
Characterization of AOS. **A** The composition of AOS was identified by TLC assay. **B** Size exclusion chromatography was performed using an ÄKTA purifier system. The prepared alginate oligosaccharides were loaded onto a Sephadex G-15 column monitored at 235 nm, which was eluted with 0.2 M ammonium bicarbonate at a flow rate of 0.3 ml/min. **C** Schematic representation of the molecular structure of AOS and sulfated AOS (AOS-SO_4_) prepared by enzymatic degradation.

The thin-layer chromatography (TLC) assay and the size exclusion chromatography assay were used to identify the composition of AOS. The TLC assay qualitatively showed the fraction distribution (Fig. 1A). The molecular weight marker was made by the lab, which was confirmed by mass spectrometry. The resulting AOS fraction distribution analysis showed that the product contains tetramers (13.3%), trimers (58.1%), and dimers (28.6%), which was determined by size exclusion chromatography [31], as shown in Fig. 1B. The sulfur content of AOS-SO_4_ is ~11.77% (w/w), which was determined using the oxygen flask combustion-chemical titration method. A schematic representation of the molecular structure of AOS and AOS-SO_4_ prepared by enzymatic degradation is shown in Fig. 1C.

### AOS-SO4 has better antitumor activity in osteosarcoma cells in vitro

**Fig. 2:**
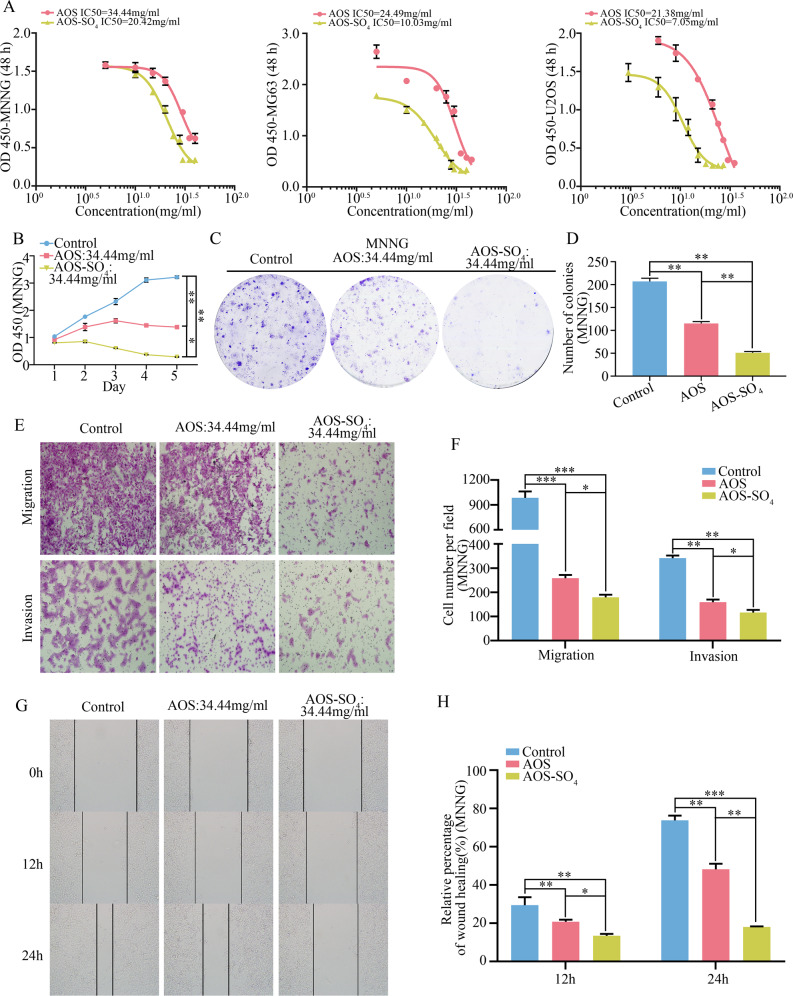
AOS-SO_4_ has better antitumor activity in osteosarcoma cells in vitro. **A** In vitro osteosarcoma cells were treated with AOS and AOS-SO_4_ at different concentrations (the range was from 0 to 45 mg/ml) for 48 h, and the IC50 was calculated. **B** A CCK-8 assay was used to detect the proliferation of osteosarcoma cells after treatment with AOS and AOS-SO_4_. **C, D** Colony formation assays in osteosarcoma cells after treatment with AOS and AOS-SO_4_ (magnification, ×40). **E, F** Transwell migration and invasion assays in osteosarcoma cells after treatment with AOS and AOS-SO_4_ (magnification, ×200). **G, H** A wound-healing assay was used to determine cell migration after treatment with AOS and AOS-SO_4_, and the wound migration distance was calculated (magnification, ×200). Data are shown as the means ± SD from at least three independent experiments. Statistical analysis was performed using Student’s *t*-test. Error bars represent the SEM. **P < 0.01; ***P < 0.001.

We investigated the growth inhibitory effect of AOS and AOS-SO_4_ against osteosarcoma cells (MNNG, MG63, and U2OS) in vitro. The half-maximal inhibitory concentration (IC50) values of AOS and AOS-SO_4_ at 48 h in the three cell lines were analyzed by GraphPad Prism 8 (Fig. 2A). For AOS, the IC50 values were 34.44 mg/ml, 24.49 mg/ml, and 21.38 mg/ml; for AOS-SO_4_, they were 20.42 mg/ml, 10.03 mg/ml, and 7.05 mg/ml (MNNG, MG63, and U2OS, respectively). Then, we used the same concentration of AOS (34.44 mg/ml) to compare the antiproliferative capacity of both compounds. The results showed that AOS and AOS-SO_4_ significantly inhibited the growth of osteosarcoma cells and the effect of AOS-SO_4_ was more effective than AOS (Fig. 2B; Supplementary Fig. 1, A, and B). Similarly, findings from the colony formation assay showed that AOS-SO_4_ more attenuated the formation of cell colonies than AOS (Fig. 2C and D; Supplementary Fig. 1C–E). Furthermore, transwell assays and wound-healing assays were conducted to determine whether AOS and AOS-SO_4_ inhibited the migration and invasion of tumor cells. AOS and AOS-SO_4_ significantly suppressed the migration and invasion of osteosarcoma cells and the effect of AOS-SO_4_ was more effective than AOS (Fig. 2E and F; Supplementary Fig. 1, F–I), and the wound gap was wider in the AOS-SO_4_ treatment group in MNNG cells (Fig. 2G and H). All these results suggested that osteosarcoma cells displayed effectiveness against AOS and AOS-SO_4_ in a concentration-dependent manner. More importantly, AOS-SO_4_ had a lower IC50 and better antitumor activity than AOS. This result demonstrated that sulfated modification of AOS exhibited a stronger antitumor effect in growth and metastasis in vitro.

### AOS-SO_4_ affects the osteosarcoma cell cycle and apoptosis in vitro and inhibits osteosarcoma cell growth and metastasis in vivo

**Fig. 3:**
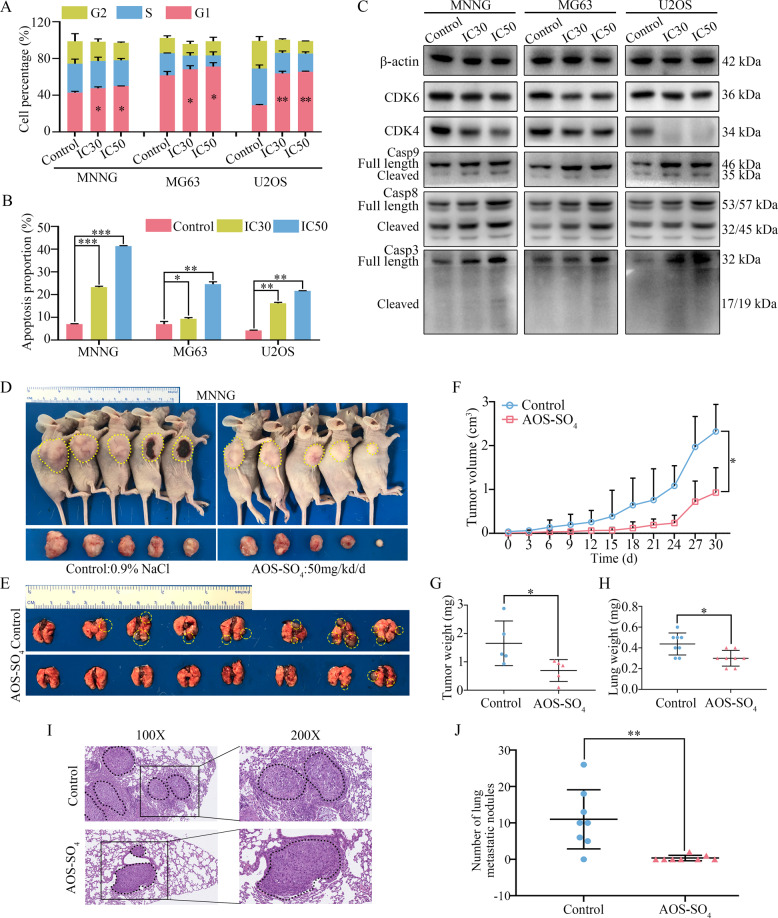
AOS-SO_4_ affects the osteosarcoma cell cycle and apoptosis in vitro and inhibits osteosarcoma cell growth and metastasis in vivo. **A** Flow cytometry histograms of the cell cycle in each phase after treatment with AOS-SO_4_ showing G1 phase arrest. **B** Flow cytometry histograms of cell apoptosis distribution after treatment with AOS-SO_4_ for 48 h. **C** After treatment with AOS-SO_4_, proteins were extracted from three cultured osteosarcoma cells and probed with appropriate dilutions of specific antibodies. Representative results of CDK4, CDK6, full length and cleavage of caspase‐9, ‐8, and ‐3 protein levels were determined by western blot analysis. **D** Photograph of tumor-bearing mice (injected by MNNG cell) and photograph of tumors after the mice were euthanized. **E** Lung metastasis formed by MNNG cells in the AOS-SO_4_ treatment and control groups. **F** Growth curve drawn by measuring tumor volumes on the indicated days. **G** Diagram showing the tumor weights in the subcutaneous tumor model. **H** Diagram showing the lung weights in the control group and AOS-SO_4_ treatment group. **I** HE staining of lung samples obtained from nude mice after injection with MNNG cells. **J** Statistical analysis of lung metastasis nodules in the AOS-SO_4_ and control groups. Data are shown as the means ± SD from at least three independent experiments. Statistical analysis was performed using Student’s *t*-test. Error bars represent the SEM. *P < 0.05; **P < 0.01; ***P < 0.001.

Next, we focused on the antitumor activity of AOS-SO_4_ in vitro and in vivo. The IC30 of AOS-SO_4_ at 48 h in three cell lines (MNNG: 13.27 mg/ml; MG63: 4.85 mg/ml; U2OS: 3.46 mg/ml) was calculated. First, flow cytometry was used to evaluate cell cycle distribution. As shown in Fig. 3A, osteosarcoma cells were arrested at G1 phase after treatment with the IC30 and IC50 AOS-SO_4_ for 48 h (Supplementary Fig. 2A). Annexin V‐FITC/PI double‐staining analysis showed that the percentages of apoptotic cells were dramatically increased after incubation with the IC30 and IC50 AOS-SO_4_ for 48 h (Fig. 3B; Supplementary Fig. 2B). Western blot analysis demonstrated that the expression of cyclin-dependent kinase 4 (CDK4) and cyclin-dependent kinase 6 (CDK6), which were the main cell cycle regulators in the G1 phase, was significantly downregulated after treatment with the IC30 and IC50 AOS-SO_4_ (Fig. 3C; Supplementary Fig. 2C). Meanwhile, after treatment with AOS-SO_4_, the expression of cleaved caspase‐9, ‐8, and ‐3 was increased after exposure to AOS-SO_4_ (Fig. 3C; Supplementary Fig. 2C). Then, we established a subcutaneous transplantation model and a lung metastasis model by transplanting MNNG cells into nude mice subcutaneously or injecting them into the lateral tail veins of nude mice to confirm the potential antitumor effect of AOS-SO_4_ in vivo. A week after injection, the mice were randomly divided into two groups: control (0.9% NaCl) and AOS-SO_4_ (50 mg/kg/d), followed by gavage with AOS-SO_4_ every other day for 30 days (subcutaneous transplantation model) or 45 days (lung metastasis model). The results demonstrated that AOS-SO_4_ inhibited tumor growth and lung metastasis (Fig. 3D and E). AOS-SO_4_ significantly decreased the tumor volume (Fig. 3F) and tumor weight (Fig. 3G) and had no effect on the bodyweight of the mice (Supplementary Fig. 2D) in the subcutaneous transplantation model. In addition, the lung weight of the AOS-SO_4_ group was lower due to fewer lung metastatic nodules (Fig. 3H). AOS-SO_4_ significantly decreased the number of lung metastatic nodules (Fig. 3I and J) and had no effect on the bodyweight of the mice in the lung metastasis model (Supplementary Fig. 2E). HE stained the heart, liver, spleen, lung, and kidney and the results showed that there was no toxicity in AOS-SO_4_-treated mice (Supplementary Fig. 3A). Taken together, we concluded that AOS-SO_4_ dramatically hindered the tumorigenesis and lung metastasis of osteosarcoma cells in vitro and in vivo.

### AOS-SO_4_ promotes autophagy in osteosarcoma cells

**Fig. 4:**
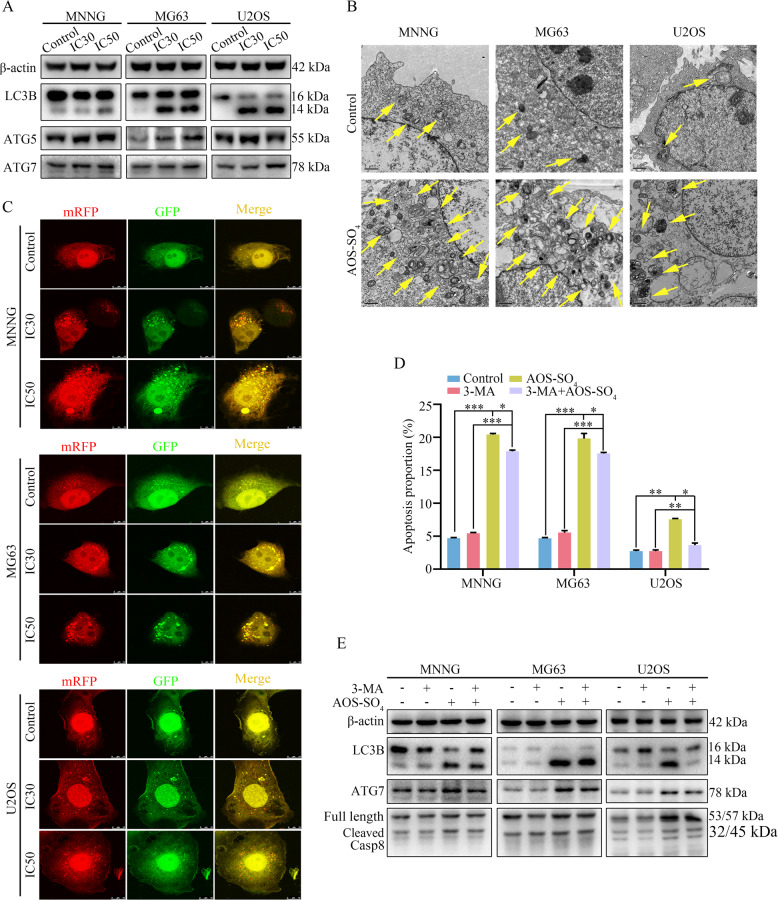
AOS-SO_4_ promotes autophagy in osteosarcoma cells. **A** After treatment with AOS-SO_4_ for 48 h, the protein levels of LC3B, ATG5, and ATG7 were assayed by western blot in osteosarcoma cells. **B** In parallel, autophagosome-like structures (indicated by the yellow arrows) were assayed by TEM. **C** LC3 puncta were analyzed by the mRFP-GFP-LC3 construct. **D** Osteosarcoma cells were treated with AOS-SO_4_ for 48 h with or without 3-MA (0.5 mM, 2 h), and flow cytometry was used to analyze apoptosis. **E** Representative blots of LC3B, ATG7, and Casp8 in MNNG, MG63, and U2OS cells after treated with AOS-SO4 for 48 h with or without 3-MA. Data are shown as the means ± SD from at least three independent experiments. Statistical analysis was performed using Student’s *t*-test. Error bars represent the SEM. *P < 0.05; **P < 0.01; ***P < 0.001.

To further elucidate the biological functions of AOS-SO_4_ in osteosarcoma, we examined the autophagy-inducing activity of AOS-SO_4_ in osteosarcoma cell lines. Autophagy-related proteins were analyzed using western blotting, and the results revealed that AOS-SO_4_ caused a significant increase in microtubule-associated proteins 1 A/1B; (LC3B), autophagy-related protein 5 (ATG5), and autophagy-related protein7 (ATG7) (Fig. 4A; Supplementary Fig. 3B). In addition, to directly demonstrate autophagosome formation, we used transmission electron microscopy (TEM) to observe numerous large autophagic vacuoles in the cytoplasm, in which the vacuolar contents were degraded, providing evidence for the impact of AOS-SO_4_ in the regulation of autophagic formation in osteosarcoma after treatment with the IC30 and IC50 of AOS-SO_4_ (Fig. 4B). Next, we utilized the mRFP-GFP-LC3 adenovirus construct to further confirm autophagy induction by forming puncta. In the present study, after infection with the mRFP-GFP-LC3 adenovirus, we observed the successful introduction of this adenovirus, showing both fluorescent proteins (Fig. 4C; Supplementary Fig. 3C). The numbers of yellow and free red puncta were both significantly higher after treatment with AOS-SO_4_, indicating increased autophagosomes and autolysosomes. In addition, the autophagy inhibitor 3-methyladenine (3-MA) was applied to osteosarcoma cells, and AOS-SO_4_-induced apoptosis was blocked by 3-MA (Fig. 4D; Supplementary Fig. 3D). As shown in Fig. 4E and Supplementary Fig. 3E, 3-MA diminished LC3B, ATG7, and cleavage of caspase‐8 induced by AOS-SO_4_. Therefore, these data suggested that AOS-SO_4_ promoted autophagy in osteosarcoma cells.

### AOS-SO_4_ promotes autophagy and induces apoptosis through the inactivation of the MEK1/ERK/mTOR signaling pathway

**Fig. 5:**
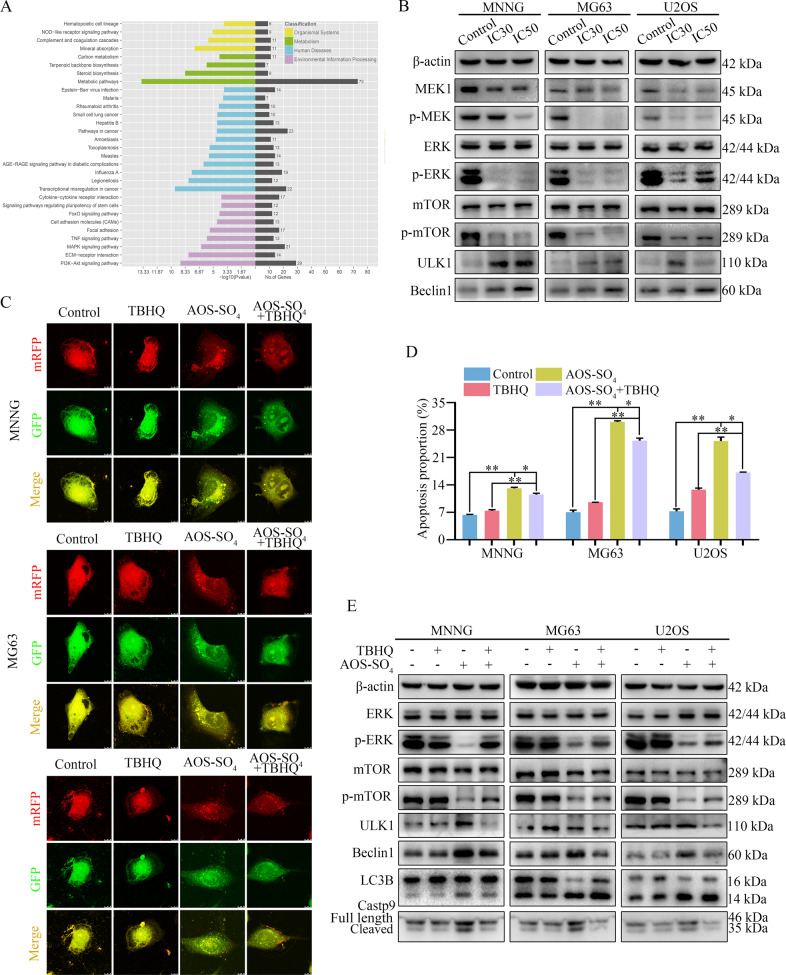
AOS-SO_4_ promotes autophagy and induces apoptosis through the MEK1/ERK/mTOR signaling pathway. **A** KEGG pathway analysis of the differentially expressed genes in AOS-SO_4_-treated MNNG cells and control cells. **B** Representative blots showing the protein expression of MEK1, p-MEK1 ERK, p-ERK, mTOR, p-mTOR, and LC3B after treatment with AOS-SO_4_. **C** Osteosarcoma cells were treated with AOS-SO_4_ for 48 h with or without TBHQ, and LC3 puncta were analyzed by the mRFP-GFP-LC3 construct. **D** Apoptosis was assessed by flow cytometry analysis after treatment with AOS-SO_4_ for 48 h with or without TBHQ. **E** Representative blots of p-ERK, p-mTOR, ULK1, Beclin1, LC3B, and Casp9 in MNNG, MG63, and U2OS cells after treated with AOS-SO4 for 48 h with or without TBHQ. Data are shown as the means ± SD from at least three independent experiments. Statistical analysis was performed using Student’s *t*-test. Error bars represent the SEM. *P < 0.05; **P < 0.01.

To explore the underlying signaling pathway affected by AOS-SO_4_, RNA-seq analysis was performed in MNNG cells. The volcano plot analysis revealed that 757 upregulated and 1083 downregulated genes were identified and quantified (Supplementary Fig. 4A). Then, 16 upregulated genes and 11 downregulated genes were chosen to verify the results of the RNA-seq analysis, and the changes were shown in the heat map (Supplementary Fig. 4, B and C). Next, Kyoto Encyclopedia of Genes and Genomes (KEGG) pathway analysis was performed based on RNA-Seq data. The results showed that AOS-SO_4_ was related to the MAPK signaling pathway, which plays a pivotal role in autophagy (Fig. 5A). Additionally, combined with mTOR, the MEK/ERK/mTOR signaling pathway has been recognized as a central signaling pathway coordinating autophagy. Next, we examined the changes in key node proteins of this pathway and autophagy-related proteins by western blotting. The results showed that the proteins p-MEK, p-ERK, and p-mTOR were significantly downregulated, and the autophagy-related proteins unc-51-like kinase 1 (ULK1) and Beclin1 were upregulated (Fig. 5B; Supplementary Fig. 4D). Moreover, we used the ERK activator tert-butylhydroquinone (TBHQ) to determine the role of MEK1/ERK/mTOR signaling in AOS-SO_4_-mediated antitumor activity. When TBHQ was combined with AOS-SO_4_, autophagic flux analysis showed that the numbers of yellow and free red puncta were both decreased (Fig. 5C; Supplementary Fig. 5A), and flow cytometry showed that apoptosis was reduced (Fig. 5D; Supplementary Fig. 5B). The expression of p-ERK, p-mTOR, ULK1, Beclin1, LC3B, and cleavage of caspase‐9 was significantly restored (Fig. 5E; Supplementary Fig. 5C). These results implied that TBHQ treatment restored AOS-SO_4-_induced cell autophagy and apoptosis. Thus, AOS-SO_4_ promoted cell autophagy and induced apoptosis through the inactivation of the MEK1/ERK/mTOR pathway.

### AOS-SO_4_ suppresses osteosarcoma cell growth through the MEK1/ERK/mTOR signaling pathway in vivo

**Fig. 6:**
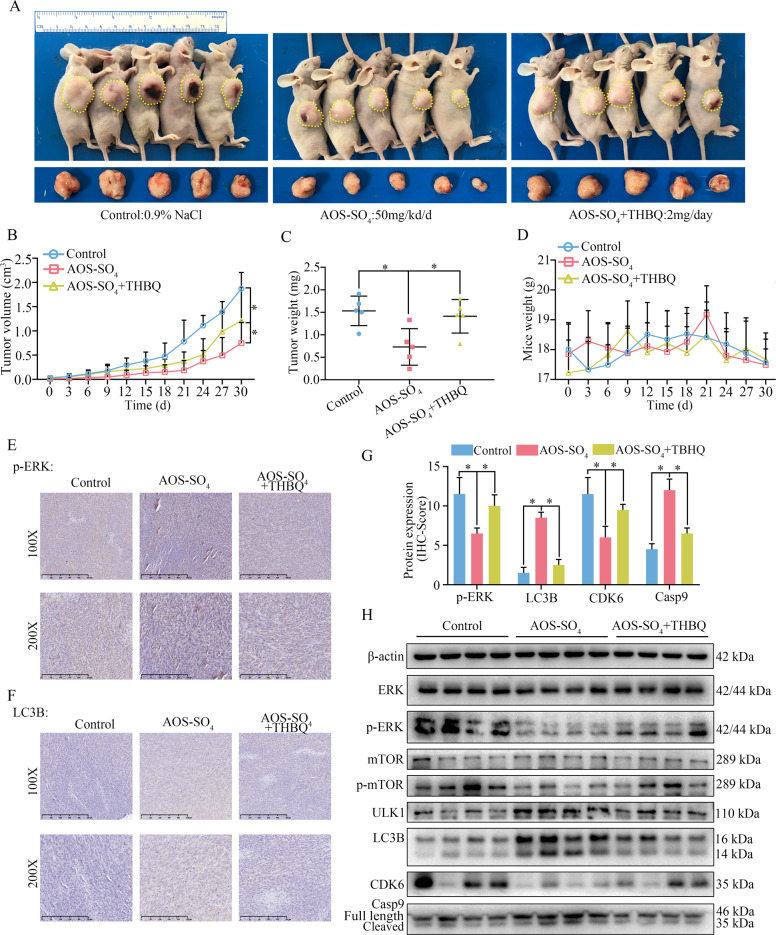
AOS-SO_4_ inhibits osteosarcoma cell growth through the MEK1/ERK/mTOR pathway in vivo. **A** Photograph of tumor-bearing mice and photograph of tumors after the mice were euthanized in the control group, AOS-SO_4_ group and AOS-SO_4_ + THBQ group. **B** Growth curve drawn by measuring tumor volumes on the indicated days. **C** Diagram showing the tumor weights in the subcutaneous tumor model. **D** Diagram showing the mouse weight in the subcutaneous tumor model. **E, F** Representative images of p-ERK and LC3B staining in the subcutaneous tumor model. **G** The quantification of IHC staining about the protein expression of p-ERK, LC3B, CDK6, and Casp9. **H** Protein levels of proliferation, apoptosis, autophagy, and phosphorylated ERK and mTOR in tumor tissues in the three groups. Statistical analysis was performed using Student’s t-test. Error bars represent the SEM. *P < 0.05.

According to the previous description, we established a tumor xenograft model by transplanting MNNG cells into nude mice subcutaneously. A week later, the mice were randomly divided into three groups: control (0.9% NaCl), AOS-SO_4_ (50 mg/kg/d), and AOS-SO_4_ (50 mg/kg/d) + TBHQ (2 mg/d), followed by gavage every other day for 30 days. The results demonstrated that TBHQ treatment could restore the tumor growth inhibited by AOS-SO_4_ (Fig. 6A). TBHQ significantly restored the tumor volume and tumor weight and had no effect on the bodyweight of the mice (Fig. 6B–D). Next, three groups of specimens were fixed in formalin for IHC staining, and four samples of each group were submerged in liquid nitrogen for cryo-etching for western blot analysis. The immunohistochemistry (IHC) analysis indicated that CDK6 and p-ERK were lower and that the staining of LC3B and caspase-9 was higher in the AOS-SO_4_ group than in the control group. TBHQ treatment restored these results (Fig. 6E–G; Supplementary Fig. 5, D and E). In addition, western blot results showed that CDK6, p-ERK, and p-mTOR proteins were markedly downregulated; ULK1, LC3B, and cleavage of caspase‐9 were upregulated in the AOS-SO_4_ group compared with the control (Fig. 6H; Supplementary Fig. 6A). Equally, the TBHQ restored these results. Taken together, we concluded that AOS-SO_4_ suppressed osteosarcoma cell growth and promoted autophagy through the MEK1/ERK/mTOR signaling pathway in vivo.

### KSR1 interacts with MEK1 in osteosarcoma cells and is a prognostic marker for osteosarcoma

**Fig. 7:**
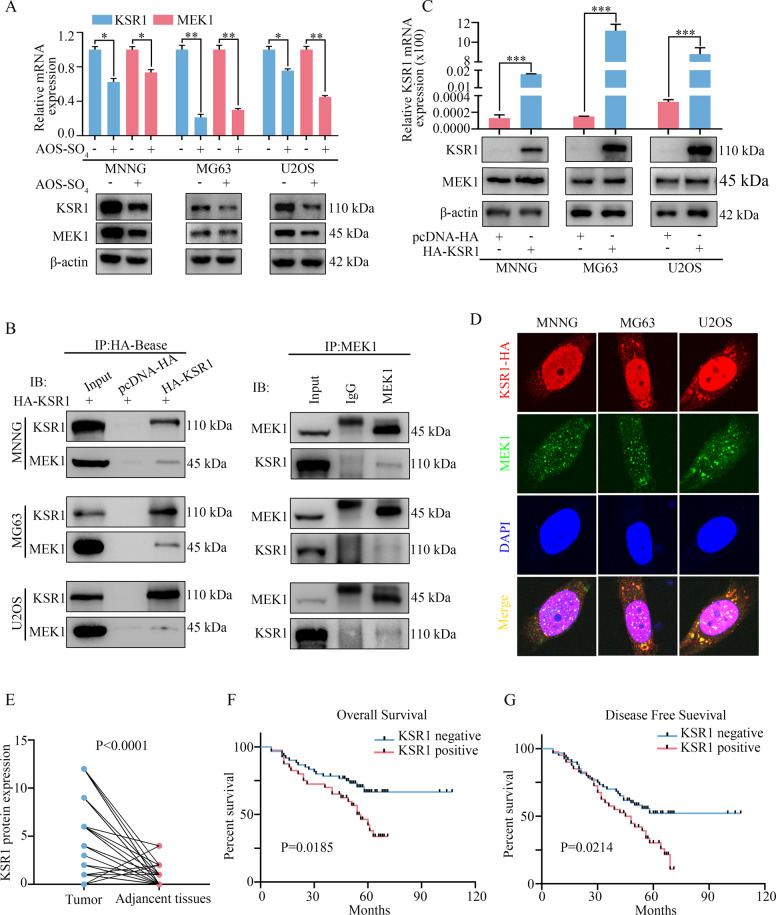
KSR1 interacts with MEK1 in osteosarcoma cells and is a prognostic marker for osteosarcoma. **A** The relative mRNA and protein expression levels of KSR1 and MEK1 after treatment with AOS-SO_4_. **B** Immunoprecipitation was carried out using anti-HA antibody and anti-MEK1 antibody, and specific associations between KSR1 and MEK1 were analyzed by western blot in osteosarcoma cells transfected with plasmids encoding HA-tagged KSR1. **C** The relative KSR1 mRNA expression and the KSR1 and MEK1 protein expression were detected after transfection with the pcDNA 3.1-HA-KSR1 plasmid. pcDNA 3.1-HA was used as a negative control. **D** Immunofluorescence analysis was performed using anti-HA and anti-MEK1 antibodies. DAPI was used as a control for nuclear staining. **E** KSR1 protein expression in 100 pairs of osteosarcoma tissues and adjacent nontumor tissues. **F, G** The effects of KSR1 expression level on overall survival and disease-free survival of patients with osteosarcoma. Data are shown as the means ± SD from at least three independent experiments. Statistical analysis was performed using Student’s *t*-test (**A, C, E**) and Log-rank test (**F, G**). Error bars represent the SEM. *P < 0.05, **P < 0.01; ***P < 0.001.

To further determine how AOS-SO_4_ regulated the MEK1/ERK/mTOR pathway in osteosarcoma, the protein interacting with MEK1 was searched in PubMed. Several researchers have reported that the kinase suppressor of ras 1 (KSR1) interacts with MEK1 and that KSR1 also plays a critical role in autophagy formation. Meanwhile, the STRING website revealed that KSR1 interacted with MEK1/2 (https://www.string-db.org/). Therefore, we detected the mRNA and protein expression of KSR1 and MEK1 after AOS-SO_4_ treatment. The results revealed that the mRNA and protein expression of KSR1 and MEK1 were significantly decreased (Fig. 7A). To further determine the interaction of KSR1 and MEK1, we constructed a pcDNA 3.1-HA-KSR1 plasmid. Coimmunoprecipitation Co-IP and western blot analysis were performed, and the results revealed that MEK1 coimmunoprecipitated with the KSR1 protein in osteosarcoma cells (Fig. 7B). In addition, after transfection with HA-tagged KSR1 in osteosarcoma cells, the mRNA and protein expression of KSR1 and MEK1 was significantly upregulated (Fig. 7C). Therefore, KSR1 could positively regulate the expression of MEK1 in osteosarcoma cells. Furthermore, the confocal analysis revealed that KSR1 and MEK1 were colocalized in osteosarcoma cells (Fig. 7D). These results indicated that KSR1 specifically interacted with MEK1 in osteosarcoma cells. To determine the clinicopathological significance of KSR1 in osteosarcoma patients, we performed an IHC analysis of KSR1 in a tissue microarray that included an independent set of 100 pairs of osteosarcoma tissues and adjacent nontumor tissues. The data of 100 osteosarcoma patients were shown in Supplementary Table 1. Representative IHC images of KSR1 expression were shown in Supplementary Fig. 6C. Immunohistochemical scores were scored in accordance with the percentage of positive cells under the microscope and the intensity of staining, and the results showed that the scores of KSR1 in tumor tissues were higher than the scores of KSR1 in adjacent tissues (Fig. 7E). Importantly, univariate analysis showed that KSR1 expression negatively correlated with overall survival and disease-free survival in 100 osteosarcoma patients (*P* < 0.05; Fig. 7F and G).

### KSR1 overexpression restores the induction of autophagy and inhibition of the MEK1/ERK/mTOR signaling pathway by AOS-SO_4_ treatment

**Fig. 8:**
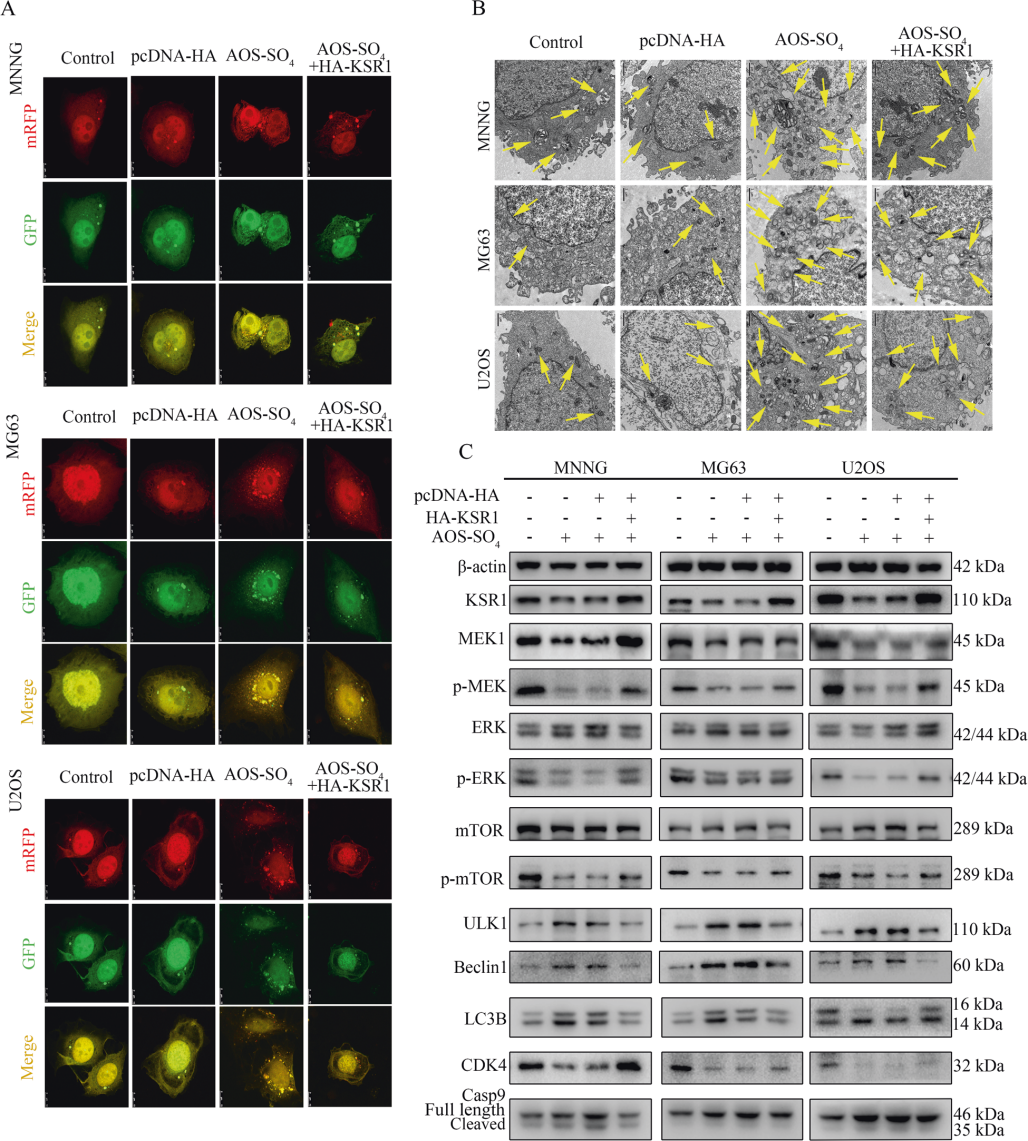
KSR1 overexpression restores the induction of autophagy and inhibition of the MEK1/ERK/mTOR signaling pathway by AOS-SO_4_ treatment. **A** Osteosarcoma cells were treated with AOS-SO_4_ for 48 h with or without transfection with HA-KSR1, and LC3 puncta were analyzed by the mRFP-GFP-LC3 construct. **B** Autophagosome-like structures (indicated by the yellow arrows) were assayed by TEM. **C** Protein levels of proliferation, apoptosis, autophagy, and KSR1, MEK1, and p-ERK in cells treated with AOS-SO_4_ for 48 h with or without transfection with the HA-KSR1 plasmid. Data are shown as the means ± SD from at least three independent experiments.

Finally, to test the role of KSR1 in AOS-SO_4_-mediated antitumor activity, we examined the activity of autophagy in HA-tagged KSR1-transfected cells after AOS-SO_4_ treatment. After infection with the mRFP-GFP-LC3 adenovirus, the numbers of yellow and free red puncta in the HA-KSR1 + AOS-SO_4_ group were both significantly lower than the numbers of yellow and free red puncta in the AOS-SO_4_ group, indicating that autophagosomes and autolysosomes decreased (Fig. 8A; Supplementary Fig. 6B). In addition, we observed that there were fewer autophagic vacuoles in the cytoplasm in the HA-KSR1 + AOS-SO_4_ group than in the AOS-SO_4_ group (Fig. 8B). Additionally, western blot analysis revealed that KSR1 overexpression restored the downregulation of MEK1, p-MEK, p-ERK, p-mTOR, and CDK4, and the upregulation of ULK1, Beclin1, LC3B, and cleavage of caspase‐9 caused by AOS-SO_4_ treatment (Fig. 8C; Supplementary Fig. 6D). These results revealed that KSR1 overexpression restores the induction of autophagy and inhibition of the MEK1/ERK/mTOR signaling pathway by AOS-SO_4_ treatment.

In conclusion, sulfated alginate oligosaccharide exerts antitumor activity and autophagy induction by inactivating MEK1/ERK/mTOR signaling in a KSR1-dependent manner in osteosarcoma.

## Discussion

Osteosarcoma is the most common type of primary bone cancer and affects children and adolescents worldwide. The most important problem is that survival rates have changed little over the past decades because no effective new drug can be used [32, 33]. AOS is a natural extract and has many biological functions, such as antitumor properties [14], regulation of immune responses [34], and reduced lipid levels [35]. AOS has been found to be widely used in the agricultural, food, and pharmaceutical industries [36]. In this study, AOS was prepared by the enzymatic degradation of alginate. Then, the oligosaccharide was sulfated to obtain AOS-SO_4_. The resulting alginate oligosaccharide fraction distribution analysis showed that the product contained tetramers (13.3%), trimers (58.1%), and dimers (28.6%), as determined by size exclusion chromatography, and the sulfurous content was ~11.77% (w/w).

AOS has been reported to be able to effectively inhibit the proliferation of osteosarcoma [15]. Sulfated derivatives of AOS have more potent antitumor activity [16, 17]. However, whether AOS-SO_4_ has better antitumor activity and the underlying mechanism have not been reported in osteosarcoma. In the current study, we first reported that AOS-SO_4_ had better antitumor activity than AOS in osteosarcoma. The flow cytometry assay demonstrated that AOS-SO_4_ induced cell cycle arrest in the G1 phase and promoted apoptosis in osteosarcoma cells. Furthermore, in subcutaneous tumors, the size and weight of the tumors formed by MNNG cells were found to be significantly decreased after treatment with AOS-SO_4_. In addition, in the lung metastasis model, there was a significant decrease in lung metastasis nodules in the AOS-SO_4_ group. From these results, we concluded that AOS-SO_4_ could inhibit the proliferation and metastasis and promote apoptosis of osteosarcoma in vitro and in vivo.

Accumulated data indicate that autophagy behaves as a two-edged sword in cancer, providing protection or causing damage to cells [20, 21]. Autophagy plays an adaptive role in protecting organisms from various types of pathological damage [37]. Autophagy is a highly regulated process involving several key mediators. Several autophagy-related genes (Atg) participate in this process. Beclin1 is a mammalian-specific Atg (also aided by ATG7 and ATG10) that plays an important role in autophagy and the development of tumors [38]. An additional protein participating in autophagy regulation is cytosolic microtubule-associated protein light chain 3 (LC3), which is an important marker of autophagy. During autophagy, LC3-I is hydrolyzed and converted to LC3-II [39]. Therefore, the ratio of LC3-II/LC3-I expression could reflect the level of autophagy. Our experimental results showed that the LC3-II/LC3-I ratio was upregulated, which confirmed that autophagy was promoted after AOS-SO_4_ treatment. In addition, TEM and autophagic flux experiments indicated that autophagosomes and autophagic flux were increased in AOS-SO_4_-treated osteosarcoma cells. Furthermore, we used the autophagy inhibitor 3-MA to further explore the mechanism of autophagy. 3-MA inhibits autophagy by inhibiting the formation of autophagosomes. After adding 3-MA, LC3B and Atg7 expressions were downregulated compared with AOS-SO_4_ treatment alone. Notably, the apoptosis rate of the AOS-SO_4_ + 3-MA group decreased synchronously, possibly because autophagy enhanced caspase-dependent cell death and induced cell death. The 3-MA inhibited autophagy from the initial stage, thus reducing cell death.

To further explore the underlying mechanism, RNA-seq analysis and bioinformatics analysis revealed that AOS-SO_4_ was related to the MAPK signaling pathway. The MAPK/mTOR pathway is commonly known to be related to autophagy and apoptosis [40, 41]. And, our results showed that AOS-SO_4_ inhibited the MEK1/ERK/mTOR signaling pathway. The MEK/ERK/mTOR signaling pathway is a classical pathway that not only promotes angiogenesis and cell progression but also plays an important role in various human malignant tumors [25]. To further test whether apoptosis and autophagy induced by AOS-SO_4_ were related to the MEK1/ERK/mTOR pathway, we added TBHQ (an ERK activator). The results showed that apoptosis was downregulated, and the levels of apoptosis-related proteins and autophagy-related proteins were decreased after TBHQ was added to AOS-SO_4_-treated cells. In addition, the autophagic flux was lower than the autophagic flux of AOS-SO_4_ treatment alone. Similarly, AOS-SO_4_ inhibited osteosarcoma cell growth through the MEK1/ERK/mTOR pathway in vivo. All these findings indicated that AOS-SO_4_ exerted antitumor activity through the MEK1/ERK/mTOR signaling pathway.

To further determine how AOS-SO_4_ regulated the MEK1/ERK/mTOR pathway in osteosarcoma, we searched related literature and the STRING website and reported that MEK1 interacted with KSR1, which is also an important autophagy regulator. KSR1 is a scaffold protein of the Raf/MEK/ERK pathway [42]. KSR1 plays an important role in regulating several cellular mechanisms to promote cell proliferation and survival, including participation in metabolic regulation, cell cycle re-initiation following DNA damage repair, and translational regulation of key mediators of a transformed phenotype [43, 44]. To date, the function of KSR1 in osteosarcoma has not yet been reported. In our research, Co-IP, western blot, and confocal analyses showed that KSR1 specifically interacted with MEK1 and that they were colocalized in osteosarcoma cells. In addition, the IHC analysis of KSR1 in a tissue microarray that included an independent set of 100 pairs of osteosarcoma tissues and adjacent nontumor tissues indicated that KSR1 was a prognostic marker for osteosarcoma patients. High KSR1 expression correlated with short overall survival and disease-free survival in osteosarcoma patients. Furthermore, autophagic flux, TEM, and western blot analysis demonstrated that KSR1 overexpression could restore the antitumor activity of AOS-SO_4_ in osteosarcoma cells. Therefore, the rescue assay revealed that induction of autophagy and inhibition of the MEK1/ERK/mTOR signaling pathway by AOS-SO_4_ treatment could be reversed by KSR1 overexpression, indicating that the activity of AOS-SO_4_ was KSR1-dependent.

Our results indicated that sulfated modification could enhance the antitumor activity of AOS. AOS-SO_4_ exerted antitumor effects and promoted autophagy by inactivating the MEK1/ERK/mTOR signaling pathway, which was KSR1-dependent. Our results show that AOS-SO_4_ might be a potentially novel candidate to treat osteosarcoma patients.

## Materials and methods

### Preparation and sulfation of AOS

Alginate was purchased from Shanghai Yuanye Co., Ltd. The alginate viscosity was 4500 cp at a 2% (w/v) concentration at 25 °C, and the M/G ratio of alginate was 0.6, as determined by NMR analysis. Alginate oligosaccharide was prepared by the enzymatic degradation of alginate using alginate lyase AlyB [31]. Alginate was dissolved in Tris-HCl buffer (50 mM, pH 7.5) with 200 mM NaCl at a concentration of 2% (w/v), followed by the addition of alginate lyase. Then, the reaction mixture was incubated at 25 °C. The reaction was terminated by heating at 100 °C for 5 min, and the sample was lyophilized after centrifugation. The resulting alginate oligosaccharides were evaluated by TLC assay and size exclusion chromatography. Then, oligosaccharide was sulfated following the canonical procedure [45]. Briefly, the resulting oligosaccharide was added to the reaction mixture (containing sulfur chloride in pyridine). Then, the reaction mixture was warmed to 100 °C for 1 h. Then, sodium hydroxide solution was added to the reaction mixture for neutralization. The final product was purified using a Sephadex G-15 column. The sulfurous content was determined using the oxygen flask combustion-chemical titration method [45].

### Cell viability assay

Osteosarcoma cells were incubated in 96-well plates for 24 h at a density of 5 × 10^3^ cells/well with 200 µL culture medium. Then, the cells were treated with different doses of AOS and AOS-SO_4_ (the range was from 0 to 45 mg/ml, with 5 mg/ml intervals). After 48 h, cell viability was detected by Cell Counting Kit-8 assay (CCK-8; Dojindo, Kumamoto, Japan). Ten microliters of CCK-8 working solution were added to each well for ~2 h, and the absorbance value of each well was measured at 450 nm with an enzyme-labeled instrument. Then, GraphPad Prism 8 software was used to calculate the half-maximal inhibitory concentration (IC50) of AOS and AOS-SO_4_.

### Flow cytometry analysis of apoptosis and cell cycle distribution

Cells were seeded in six-well plates (3 × 10^5^ cells/well) and then exposed to the IC30 and IC50 of AOS-SO_4_, 3-MA, and TBHQ or their combinations. After 48 h, cells were collected, washed, and stained according to the manufacturer’s guidelines (Annexin V-FITC Apoptosis Detection Kit; Beyotime, China). Then, the samples were read on a flow cytometer (BD Biosciences, Franklin Lakes, NJ, USA). For the cell cycle assay, cells were treated as mentioned above, collected according to the manufacturer’s guidelines (Cell Cycle and Apoptosis Analysis Kit; Beyotime, China), and analyzed by flow cytometry.

### Animal experiment

Female BALB/c nude mice (20 g/mouse) were used in animal studies, and all animals were maintained in specific pathogen-free (SPF) conditions. MNNG cells were injected subcutaneously into the left scapula of nude mice (1 × 10^6^ cells/mouse). The mice were randomized into the following two groups: (1) 0.9% NaCl group (0.9% NaCl in 200 µL, po/day/mouse); (2) AOS-SO_4_ group (AOS-SO_4_ 50 mg/kg/day), or three groups: (1) 0.9% NaCl group (0.9% NaCl in 200 µL, po/day/mouse); (2) AOS-SO_4_ group (AOS-SO_4_ 50 mg/kg/day); and (3) AOS-SO_4_ + TBHQ group (AOS-SO_4_ 50 mg/kg/day + THBQ 2 mg/day). For lung metastasis studies, the mice were tail vein-injected with 2 × 10^6^ MNNG cells. After 1 week, the mice were randomized into two groups as mentioned above. The body weights and tumor volumes of the mice were measured every 3 days. After 30/45 days, the mice were euthanized, and the xenograft tumors from each animal were weighed and analyzed. The lung tissues of each animal were weighed and fixed in 10% formalin for histological analysis. The fixed samples were embedded in paraffin and made into tissue sections. Some sections were stained with hematoxylin and eosin (HE), and others were stained with immunohistochemistry (IHC). All animal experiments were housed and performed according to the guidelines approved by the Shanghai Medical Experimental Animal Care Commission.

### mRFP-GFP-LC3-expressing cell generation and fluorescent LC3 puncta analysis

To perform image-based analysis for autophagy, cells were infected with tandem GFP-RFP-LC3 adenovirus for 24 h according to the manufacturer’s instructions [46]. mRFP-GFP-LC3 adenoviral vectors were purchased from HanBio Technology (Shanghai, China). Then, cells were treated and imaged for green fluorescent protein (GFP) and red fluorescent protein (RFP) by using a Fluoview FV1000 microscope (Olympus, Tokyo, Japan).

### Statistical analysis

All data were presented as the average of three independent experiments. Statistical analysis was conducted with Graghpad Prism 8 software (San Diego, CA, USA). Differences between experimental groups and control groups were calculated by Student’s t-test. The overall survival and disease-free survival of patients were calculated by the Log-rank test. Correlation analysis of KSR1 protein expression in relation to clinicopathologic was calculated by Chi-square test. And *P* < 0.05 was considered statistically significant (*P < 0.05; **P < 0.01; ***P < 0.001).

## Supplementary information

Supplemental materials

Declaration of Interest Statement

## Data Availability

All data generated or analyzed during this study are included in this published article.
