# Supplementary material for: Sulfated alginate oligosaccharide exerts antitumor activity and autophagy induction by inactivating MEK1/ERK/mTOR signaling in a KSR1-dependent manner in osteosarcoma
Source: Oncogenesis. 2022 Apr 13;11(1):16. doi: 10.1038/s41389-022-00390-x (PMC9008062; doi:10.1038/s41389-022-00390-x)
Supplement: Supplementary file 1 — Supplemental materials [file 41389_2022_390_MOESM1_ESM.pdf]

Supplementary Materials

Supplementary methods

Reagents and Antibodies. The 3-methyladenine (3-MA, S2767) and tert-butylhydroquinone (TBHQ, S4990) were purchased from Selleck (Shanghai, China). Anti-MEK (ab32576) and anti-KSR1 (ab68483) antibodies were purchased from Abcam (Shanghai, China). Anti-LC3B (3868T), Atg5 (12994T), Atg7 (8558T), mTOR (2983T), p-mTOR (5536T), ERK (4695T), p-ERK (4370T) and p-MEK (2338T) were purchased from Cell Signaling Technology (CST, Shanghai, China). Anti-β-actin (HRP-60008), CDK4 (66950-1-Ig), CDK6 (14052-1-AP), MEK1 (11049-1-AP), ULK1 (20986-1-AP), Beclin1 (66665-1-Ig), HA-Tag (66006-2-Ig), Caspase 9 (10380-1-AP), Caspase 8 (66093-1-Ig), and Caspase 3 (66470-2-Ig) were purchased from Proteintech (Wuhan, Hubei, China).

CCK-8 assay. Osteosarcoma cells were seeded in 6-well plates (3×10^5 cells/well) and treated with the IC50 of AOS and AOS-SO_4 for 48 h. The cells were digested and seeded in 96-well plates (5000 cells/well), treated with culture medium supplemented with 10% FBS and evaluated by CCK-8 assay for 5 days.

Cell Culture. Human osteosarcoma cell lines (MNNG, MG63, and U2OS) were obtained from the Cell Bank of the Chinese Academy of Sciences (Shanghai, China), and they were recently authenticated and tested for mycoplasma contamination. Cells were raised in Dulbecco's modified Eagle's medium (DMEM) or RPMI-1640 medium supplemented with 10% fetal bovine serum (Gibco, Grand Island, NY, USA), 1% penicillin-streptomycin and 1% nonessential amino acids (NEAAs) under standard culture conditions (37°C, 95% humidified air and 5% CO_2). The medium was changed every 2 days.

Colony formation assay. After treatment with the IC50 of AOS and AOS-SO_4 for 48 h, cells were cultured in 6-well plates at a density of 1000 cells/well and incubated under standard culture conditions for 10 days. Next, the medium was removed, and the cell clones were washed with PBS. Then, the cell

clones were fixed with 4% paraformaldehyde and dyed with 0.1% crystal violet. Finally, the cell colonies were counted.

Cell migration and invasion assays. Cell migration and invasion ability were assessed through transwell filter chambers (Corning, New York, NY, USA), which were 8-mm pore size chamber inserts in a 24-well plate. For the migration assay, 48 h after the IC50 of AOS and AOS-SO_4 treatment, 200 μL of serum-free medium containing 5×10^4 MNNG and U2OS cells or 1×10^5 MG63 cells was dropped into the upper chambers. For the invasion assay, 1×10^5 MNNG, U2OS cells or 2×10^5 MG63 cells were placed into the upper chambers, which were coated with Matrigel diluted with serum-free culture medium. Separately, 800 μL of culture medium supplemented with 10% FBS was added to the lower chambers. After incubation at 37°C, the cells on the bottom surface of the membrane were stained and counted.

Wound-healing assay. Osteosarcoma cells were seeded in 6-well plates to reach 90% confluence. Then, the wounds were scraped/wounded using a yellow tip, treated with different drugs (the IC50 of AOS and AOS-SO_4) and measured at 0 h, 12 h, and 24 h by microscopy.

Western blot analysis. The tumor tissue was weighed and added to RIPA lysis buffer (Thermo Fisher, Waltham, USA) at a ratio of 1:8 (1 g tissue plus 8 ml RIPA lysis buffer). Protease and phosphatase inhibitors were added at a ratio of 100:1. The tissue was homogenized with a homogenizer on ice, mixed and sucked into an EP tube. In addition, cells were scraped into EP tubes with cell scraping tools and mixed with RIPA lysis buffer. All operations were carried out on ice. After measuring the protein concentration with BCA protein assays (Thermo Fisher, Waltham, USA), 5× SDS loading buffer was added to each EP tube and heated at 95°C for 5 min. Proteins were separated by 8%-12% SDS-PAGE and transferred to polyvinylidene fluoride (PVDF) membranes (Millipore, Massachusetts, USA). The

protein mass marker was a mixture of proteins from 10 kDa to 245 kDa (Yeasen, Shanghai, China, 20352ES76). The membranes were incubated with 1:1000-5000 primary antibodies at 4°C overnight, followed by incubation with secondary antibodies at room temperature for 1 h. Finally, an ECL detection system (SmartChemi 420, Beijing, China) was used to measure the immune reaction zone. Western blot images were quantified using ImageJ software.

Cell transfection and efficiency test. Lipofectamine 2000 (Invitrogen, California, USA) was used for plasmid transfection. The pcDNA3.1-HA-KSR1 plasmid was designed and synthesized by GENEWIZ (Suzhou, China). Cells were plated at 60-70% confluence in a 6-well plate and transfected with pcDNA3.1-HA or pcDNA3.1-HA-KSR1 using Lipofectamine 2000 Reagent according to the manufacturer's protocol. The transfection efficiency was determined by qRT-PCR and Western blot.

RNA extraction and real-time polymerase chain reaction (RT-PCR) assay. Total RNA was extracted with TRIzol reagent (Invitrogen, California, USA) according to the manufacturer's protocol. First-strand cDNA was synthesized with a PrimeScript RT Reagent Kit (Takara, Dalian, China). The cDNA templates were combined with SYBR Green premix with Rox II (Takara, Dalian, China) to perform quantitative real-time polymerase chain reaction (qRT-PCR). Information on the primer sequences used was presented in Supplementary Table 2.

Coimmunoprecipitation. Coimmunoprecipitation was performed in osteosarcoma cells. Equal amounts of protein (3000 μg) were incubated with antibodies at 4 °C overnight followed by incubation with protein A/G magnetic beads (Biotool) for 3 h at 4 °C. The beads were washed using phosphate-buffered saline (PBS) containing 1% Triton X-100. IgG-bound, HA-bound or MEK1-bound proteins were separated using SDS-PAGE for Western blot analysis.

Confocal immunofluorescence. Confocal immunofluorescence was performed on osteosarcoma cells.

Cells were fixed and incubated with a mouse monoclonal anti-HA antibody and a rabbit polyclonal anti-MEK1 antibody overnight at 4 °C followed by 1 h incubation with secondary antibodies (Invitrogen). The cells were incubated with a 1:1000 dilution of 4′,6-diamidino-2-phenylindole (DAPI) for 5 min and viewed with a Fluoview FV1000 microscope (Olympus).

Transmission electronic microscopy. Cells treated with or without AOS-SO_4 were harvested, fixed, dehydrated, embedded in Epon, stained and observed. Images were acquired using an HT7700 electron microscope (Hitachi, Tokyo, Japan) to observe the autophagic vacuoles. The number of autophagic vacuoles (AVs) in each cell was quantified using 20 randomly selected cells of each group.

Clinical samples and IHC. The tissue microarray contained 100 patients diagnosed with osteosarcoma at Shanghai Sixth People's Hospital. They received primary surgical treatment and preoperative and postoperative neoadjuvant therapy. Ethics approval was obtained from the Ethics Committee of the Shanghai Jiao Tong University Affiliated Sixth People's Hospital, and written informed consent was obtained from each patient prior to sample collection. A statement confirmed that all methods were carried out in accordance with relevant guidelines and regulations. A standard IHC staining procedure was followed. Briefly, paraffin-embedded sections were cut at 4 μm, dewaxed in xylene, and heated in a microwave at 60 °C for 20 min in EDTA buffer (pH 9.0) for antigen retrieval. For each slide, endogenous peroxidase activity was blocked by a 10 min incubation in 0.3% H_2O_2 followed by incubation at 37 °C with a 1:100 dilution of the primary antibody KSR1. Slides were rinsed three times in PBS, incubated for 30 min with an EnVision staining kit (DAKO), followed by three additional washes in PBS, and color was developed over 3-10 min in a moist chamber at room temperature using 3,3′-diaminobenzidine. Slides were counterstained in hematoxylin and dehydrated in a graded ethyl alcohol series (70%, 90%, and 100%). Assessment of IHC staining was independently performed by

two expert pathologists. Any discordance was resolved through discussion and consensus. IHC signal intensities were scored as follows: negative, weak, moderate, or strong.

Study approval. All animal studies were conducted in compliance with protocols approved by the guidelines approved by the Shanghai Medical Experimental Animal Care Commission (Shang hai, China). And all animal experiments complied with the National Institutes of Health guide for the care and use of Laboratory animals. Clinical information was assimilated from patient records from the Shanghai Jiao Tong University Affiliated Sixth People's Hospital. Informed consent was obtained for each patient on an ongoing research protocol approved by the Ethics Committee of the Shanghai Jiao Tong University Affiliated Sixth People's Hospital (No. 2020-YS-051).

Supplementary table 1. Correlation analysis of CLTC protein expression in relation to clinicopathologic variables of 100 patients with osteosarcoma.

**Correlation analysis of CLTC protein expression in relation to clinicopathologic variables of 100 patients with osteosarcoma**			
**Clinicopathologic paremeters**	**KSR1 expression level**		**P value**
	**Negative**	**Positive**	
Gender			
Male	18	16	0.301
Female	42	24	
Age(years)			
<20	41	27	0.930
≥20	19	13	
Location			
Distal femur	36	18	0.324
Proximal tibia	13	11	

Elsewhere	11	11	
Tumor necrosis rate(%)			
<90	51	27	0.038*
≥90	9	13	
Operation type			
Amputation	8	7	0.568
Limb salvage	52	33	
Recurrence			
Yes	14	17	0.042*
No	46	23	
AJCC/TNM stage			
I/IIA	26	8	0.016*
IIB/III/IV	34	32	

**P value*: Correlation analysis of CLTC protein expression in relation to clinicopathologic were calculated by Chi-square test. And $P<0.05$ was considered statistically significant (*$P<0.05$).

Supplementary Table 2. The primer sequences for qRT-PCR

Gene name		Sequence (5'-3')
MTIG	Forward	GTGTCTCCTGCACCTGCG
	Reverse	AGGAGCAGCAGCTCTTCTTG
NOXO1	Forward	AATTCAGGCAGCTCAAGACCC
	Reverse	CGGTCTTCGTTCTCCACCAG
SLC35G5	Forward	CACAATGGCTGGAGCTCTGA
	Reverse	CAGGCAGGTTGAAGTAGGGG
LYNX1	Forward	CTGCACCAACCATCCCCTG
	Reverse	CTGGTGGGGGTGTAGTAGGT
PIWIL4	Forward	AAGCCCACACACACCTTTCA
	Reverse	TGGTCAGTCAGCCCTGTTAG
FABP4	Forward	AAACTGGTGGTGGAATGCGT

	Reverse	GCGAACTTCAGTCCAGGTCA
LEKR1	Forward	ACACCCGTCCTAGTCGAAGT
	Reverse	TTGTCCATCTCTGTCACCGC
MT1E	Forward	CGCCACTGGTGGCTCC
	Reverse	TGAGTTCCCTCCCAACCTGA
MT1X	Forward	TTGATCGGGAACTCCTGCTT
	Reverse	CAGGAGCCAACAGGCGA
OSGIN1	Forward	GTCATCATTGTGGGTAACGGC
	Reverse	CTGTGTCTGGGCGTAGAAGG
NPTX1	Forward	GGAGAACCTCGAGCAGTACA
	Reverse	TTTCTCGAGCTCGCTGATCC
HMGCS1	Forward	GGACTGTCCTTTCGTGGCTC
	Reverse	GGCATGGTGAAAGAGCTGTG
MMP1	Forward	AGAAAGAAGACAAAGGCAAGTTGA
	Reverse	TTGTCCCGATGATCTCCCCT
KRT3	Forward	GTACCAGACCAAGTTGGGGG
	Reverse	GCCGTAACCTCCTCCATAGC
EDA	Forward	ACTCGAGAAAACCAGCCAGC
	Reverse	GTCATTGAGCACTCCACCTGA
LRG1	Forward	AGACAGCGACCAAAAAGCCCA
	Reverse	ATTTCGGCAGGTGGTTGACA
IL6	Forward	CCACCGGGAACGAAAGAGAA
	Reverse	TCACCAGGCAAGTCTCCTCA
PARX6	Forward	ACTGCCGCTATGAACCACAA
	Reverse	CTACAGAGCCACCCAGCAAC
ID3	Forward	CTTCCCATCCAGACAGCCGA
	Reverse	TCACAGTCCTTCGCTCCTGA
MYBPH	Forward	GAGAGACGGTCAACCTGCAA
	Reverse	GGGGGTCCAGGTTTCTCAAT

HMOX1	Forward	CTCCTCTCGAGCGTCCTCAG
	Reverse	AAATCCTGGGGCATGCTGTC
CYP1A1	Forward	AACCTTTGAGAAGGGCCACAT
	Reverse	TTACAAAGACACAACGCCCCT
CSF3	Forward	GGAGAAGCTGGTGAGTGAGTGTG
	Reverse	AAAGGCCGCTATGGAGTTGG
TXNIP	Forward	TCAGTATTGCAGGGCTTGGC
	Reverse	TAGCAGACACAGGTGCCATT
CXCL8	Forward	AGTTTTTGAAGAGGGCTGAGA
	Reverse	ACCAAGGCACAGTGGAACAA
ALPK2	Forward	CGGACATGCAAGGTGTAGGA
	Reverse	TTCTTTTCGCCTGGGGTCTC
TMPRSS13	Forward	CAGAAGCAGCTACCGCTCAT
	Reverse	AAGGGCATTCAGACCTGTGG
β-actin	Forward	GTCATTCCAAATATGAGATGCGT
	Reverse	GCATTACATAATTTACACGAAAGCA

Supplementary Figures

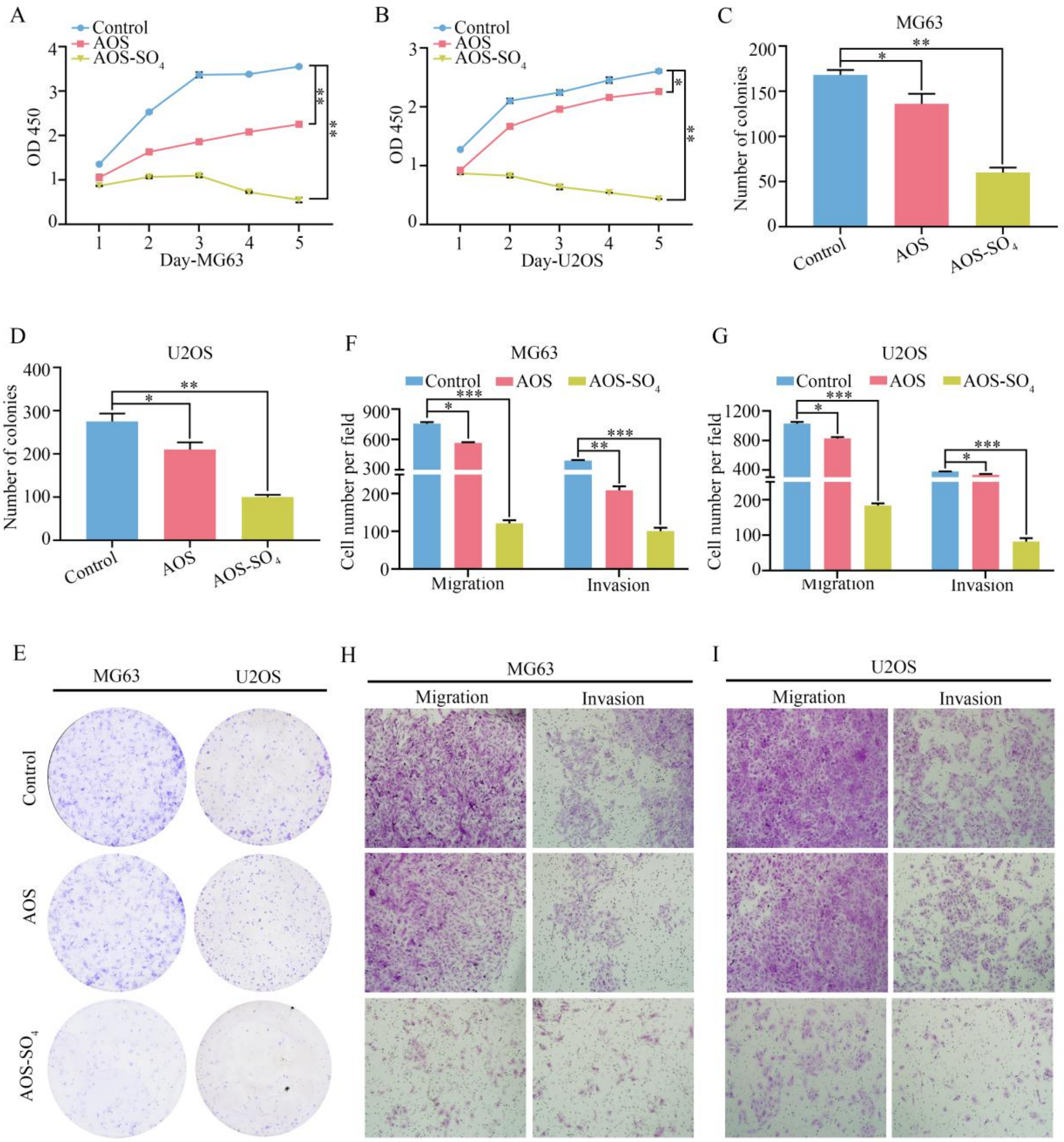

Supplementary Figure 1. (**A, B**) The CCK-8 assay was used to detect the proliferation of MG63 and U2OS cells after treatment with AOS and AOS-SO4. (**C, D, E**) Colony formation assays in MG63 and U2OS cells after treatment with AOS and AOS-SO4 (magnification, ×40). (**F-I**) Transwell migration and invasion assays in MG63 and U2OS cells after treatment with AOS and AOS-SO4 (magnification, ×200). Data are shown as the means ± SD from at least three independent experiments. Statistical analysis was performed using Student's t-test. Error bars represent the SEM. *P<0.05; ** P<0.01; *** P<0.001.

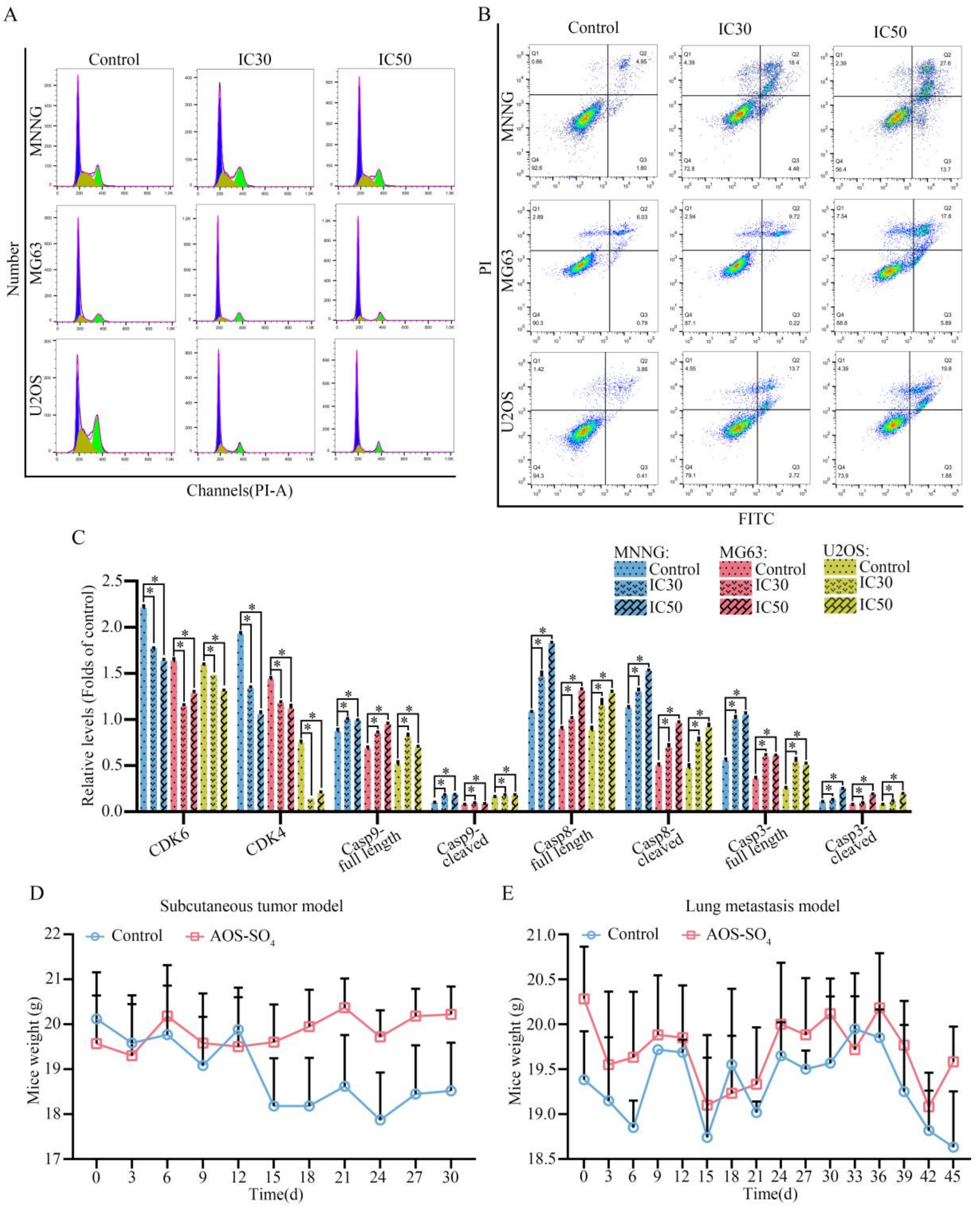

Supplementary Figure 2. (**A**) Flow cytometry of cell cycle distribution in each phase after treatment with AOS-SO_4 after 48 h showing G1 phase arrest. (**B**) Flow cytometry of cell apoptosis distribution after treatment with AOS-SO_4 for 48 h. (**C**) Protein levels of cell cycle- and apoptosis-related proteins after treatment with AOS-SO_4 for 48 h, assayed by Western blot. (**D, E**) Growth curve drawn by measuring the weight of mice on the indicated days in subcutaneous tumors and lung metastasis model. Statistical analysis was performed using Student's t-test. Error bars represent the SEM. *P<0.05.

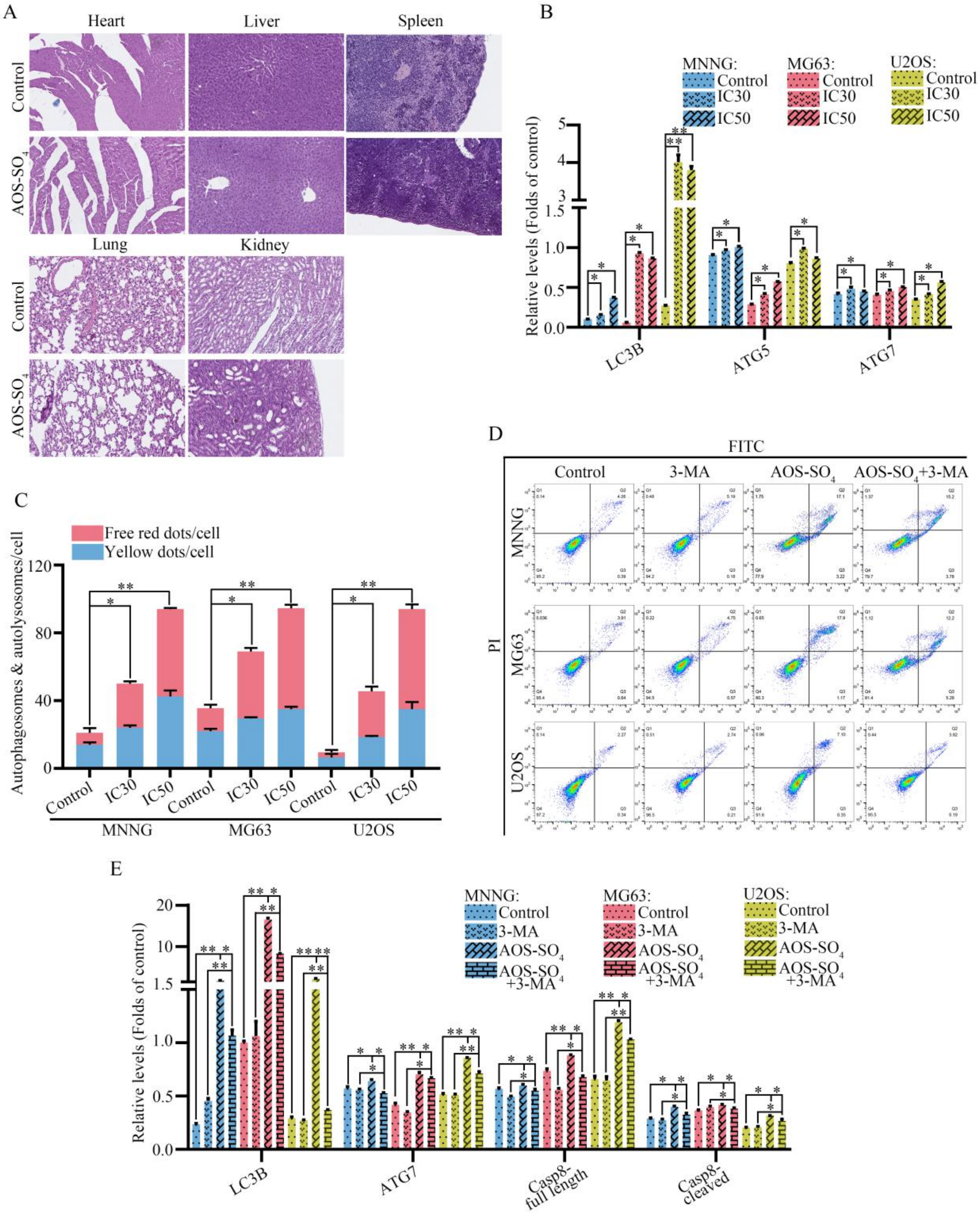

Supplementary Figure 3. (**A**) HE staining of the heart, liver, spleen, lung, and kidney organs in AOS-SO4-treated MNNG mice and control mice. (**B**) Protein levels of LC3B, ATG5 and ATG7 after treatment with the IC30 and IC50 of AOS-SO4 for 48 h, assayed by Western blot. (**C**) The LC3 puncta were analyzed by the mRFP-GFP-LC3 construct after treatment with AOS-SO4 for 48 h with or without 3-MA. (**D**) Flow cytometry was used to analyze the apoptosis change after treatment with AOS-SO4 for 48 h with or without 3-MA. (**E**) Autophagy-related proteins were analyzed by Western blot after treatment with AOS-SO4 for 48 h with or without 3-MA. Data are shown as the means ± SD from at least three independent experiments. Statistical analysis was performed using Student's t-test.

Error bars represent the SEM. *P<0.05; ** P<0.01; *** P<0.001.

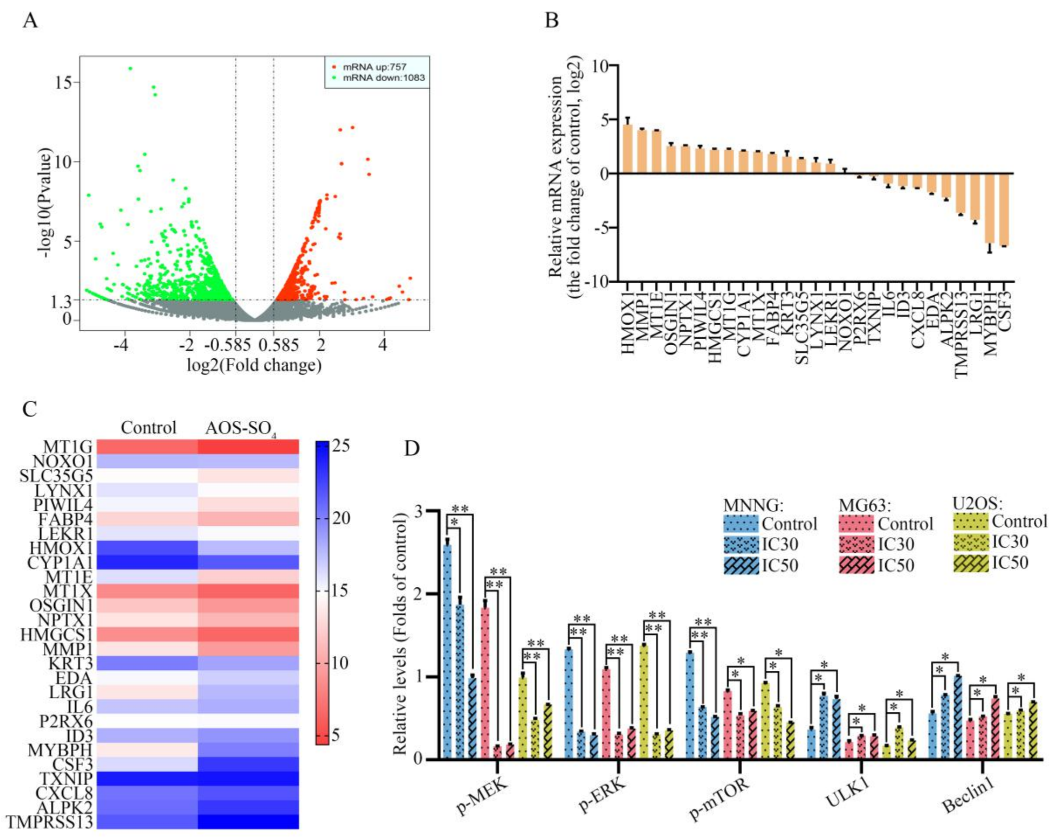

Supplementary Figure 4. (**A**) A volcano plot analysis revealed that 757 upregulated and 1083 downregulated genes were identified and quantified. (**B**) The mRNA expression levels of 16 upregulated and 11 downregulated proteins in osteosarcoma cells. (**C**) Heatmap displaying the mRNA expression levels of 16 upregulated and 11 downregulated proteins in osteosarcoma cells. (D) Western blot showing the protein expression of p-MEK1 and LC3B after treatment with AOS-SO_4. Data are shown as the means ± SD from at least three independent experiments. Statistical analysis was performed using Student's t-test. Error bars represent the SEM. *P<0.05; ** P<0.01.

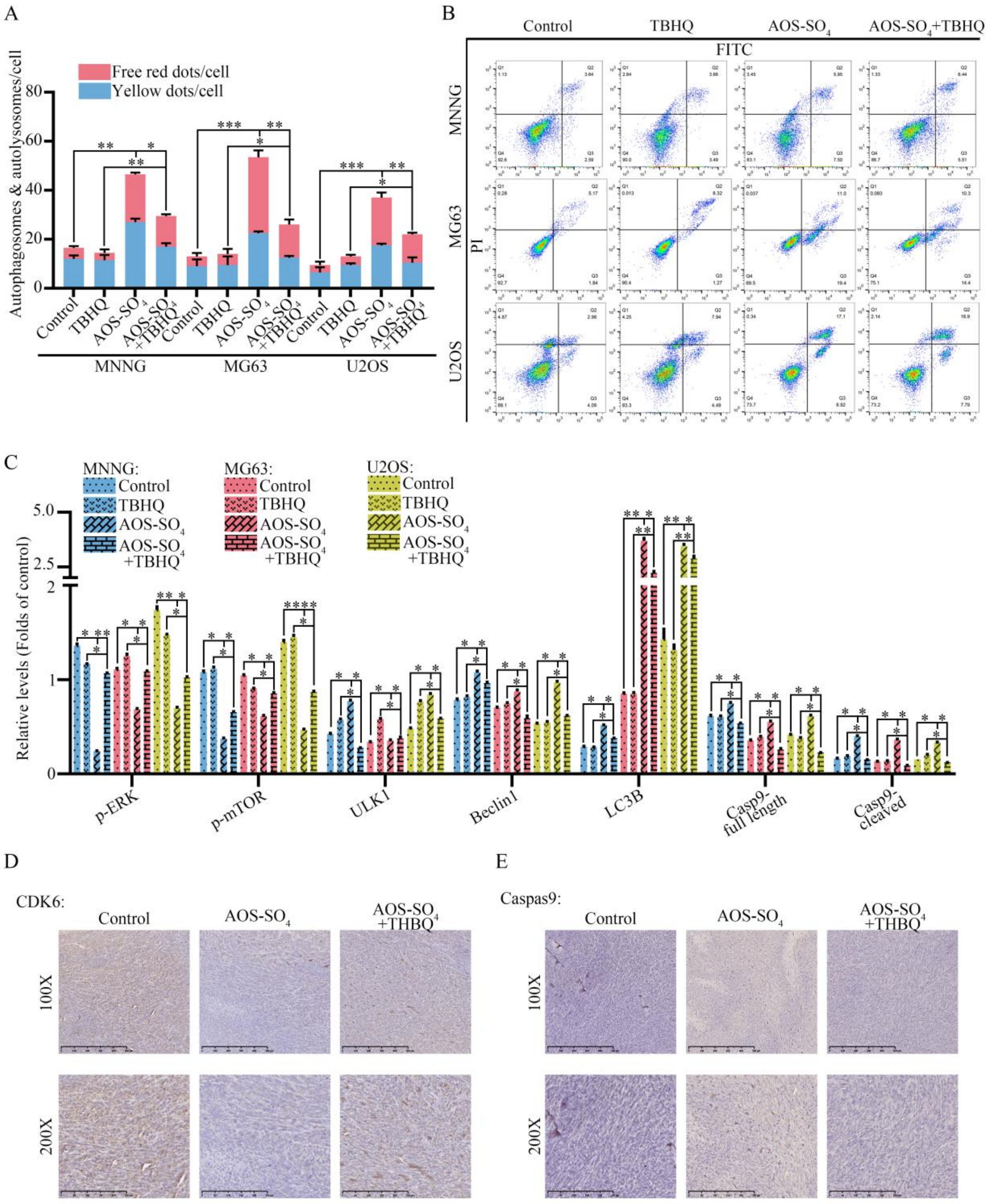

Supplementary Figure 5. (**A**) The LC3 puncta were analyzed by the mRFP-GFP-LC3 construct after treatment with AOS-SO_4 for 48 h with or without TBHQ. (**B**) Flow cytometry was used to analyze apoptosis after treatment with AOS-SO_4 for 48 h with or without TBHQ. (**C**) The relative protein levels of p-ERK, p-mTOR and LC3B after treatment with AOS-SO_4 for 48 h with or without TBHQ. (**D, E**) Representative images of CDK6 and Caspase-9 staining in the subcutaneous tumor model. Data are shown as the means ± SD from at least three independent experiments. Statistical analysis was performed using Student's t-test. Error bars represent the SEM. **P*<0.05; ** *P*<0.01; *** *P*<0.001.

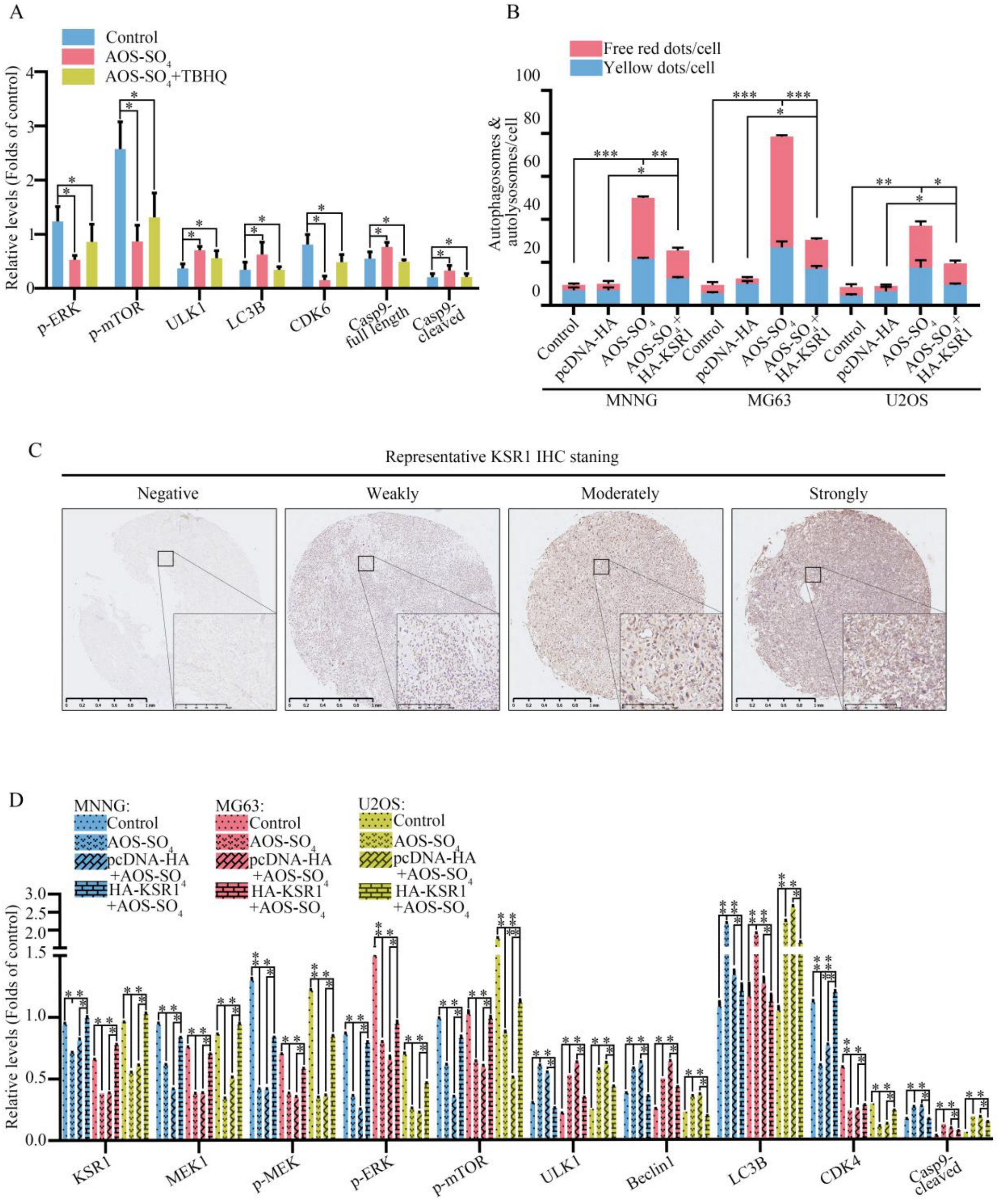

Supplementary Figure 6. (**A**) The relative protein levels of p-ERK, p-mTOR and LC3B in the subcutaneous tumor model. (**B**) The LC3 puncta were analyzed by the mRFP-GFP-LC3 construct after treatment with AOS-SO_4 for 48 h with or without transfection with the HA-KSR1 plasmid. (**C**) Representative IHC images of KSR1 in osteosarcoma tissues. IHC signal intensity scale: negative, weak, moderate and strong. Original magnification: 50×, 200×. (**D**) The relative protein levels of KSR1, MEK1, p-ERK, p-mTOR and LC3B after treatment with AOS-SO_4 for 48 h with or without transfection with the HA-KSR1 plasmid. Data are shown as the means ± SD from at least three independent experiments. Statistical analysis was performed using Student's t-test. Error bars represent

the SEM. *P<0.05; ** P<0.01.
