# Supplementary material for: Sulfated alginate oligosaccharide exerts antitumor activity and autophagy induction by inactivating MEK1/ERK/mTOR signaling in a KSR1-dependent manner in osteosarcoma
Source: Oncogenesis. 2022 Apr 13;11(1):16. doi: 10.1038/s41389-022-00390-x (PMC9008062; doi:10.1038/s41389-022-00390-x)
Supplement: Supplementary file 2 — Declaration of Interest Statement [file 41389_2022_390_MOESM2_ESM.pdf]

Declaration of Interest Statement：

On behalf of all authors, I would like to declare that there is no conflict of interest exits in the submission of this manuscript, and manuscript is approved by all authors for publication.

Qing-cheng Yang, M.D.
Department of Orthopedics,
Shanghai Jiao Tong University Affiliated Sixth People’s Hospital
E-mail: tjyqc@163.com
